# Microwave-Assisted Synthesis of Modified Glycidyl Methacrylate–Ethyl Methacrylate Oligomers, Their Physico-Chemical and Biological Characteristics

**DOI:** 10.3390/molecules27020337

**Published:** 2022-01-06

**Authors:** Adam Chyzy, Damian Pawelski, Vladyslav Vivcharenko, Agata Przekora, Michael Bratychak, Olena Astakhova, Joanna Breczko, Pawel Drozdzal, Marta E. Plonska-Brzezinska

**Affiliations:** 1 Department of Organic Chemistry, Faculty of Pharmacy with the Division of Laboratory Medicine, Medical University of Bialystok, Mickiewicza 2A, 15-222 Bialystok, Poland; adam.chyzy@umb.edu.pl (A.C.); damian.pawelski@umb.edu.pl (D.P.); 2 Independent Unit of Tissue Engineering and Regenerative Medicine, Medical University of Lublin, Chodzki 1, 20-093 Lublin, Poland; vlad.vivcharenko@gmail.com (V.V.); agata.przekora@umlub.pl (A.P.); 3 Department of Petroleum and Gas Chemistry and Technology, Lviv Polytechnic National University, 12, St. Bandera Str., 79013 Lviv, Ukraine; mbratychak@gmail.com (M.B.); olena.brat@gmail.com (O.A.); 4 Faculty of Chemistry, University of Bialystok, Ciolkowskiego 1K, 15-245 Bialystok, Poland; j.luszczyn@uwb.edu.pl; 5 Department of Structural Biology of Prokaryotic Organisms, Institute of Bioorganic Chemistry, Polish Academy of Sciences, Z. Noskowskiego St. 12/14, 61-704 Poznan, Poland; pdrozdzal@ibch.poznan.pl

**Keywords:** microwave-assisted synthesis, hydrogel, glycidyl methacrylate, ethyl methacrylate, *co*-oligomer, biomedicine, hydrophilic matrix

## Abstract

In this study, well-known oligomers containing ethyl methacrylate (EMA) and glycidyl methacrylate (GMA) components for the synthesis of the oligomeric network [P(EMA)-*co*-(GMA)] were used. In order to change the hydrophobic character of the [P(EMA)-*co*-(GMA)] to a more hydrophilic one, the oligomeric chain was functionalized with ethanolamine, xylitol (Xyl), and L-ornithine. The oligomeric materials were characterized by nuclear magnetic resonance and Fourier transform infrared spectroscopy, scanning electron microscopy, and differential thermogravimetric analysis. In the final stage, thanks to the large amount of -OH groups, it was possible to obtain a three-dimensional hydrogel (HG) network. The HGs were used as a matrix for the immobilization of methylene blue, which was chosen as a model compound of active substances, the release of which from the matrix was examined using spectrophotometric detection. The cytotoxic test was performed using fluid extracts of the HGs and human skin fibroblasts. The cell culture experiment showed that only [P(EMA)-*co*-(GMA)] and [P(EMA)-*co*-(GMA)]-Xyl have the potential to be used in biomedical applications. The studies revealed that the obtained HGs were porous and non-cytotoxic, which gives them the opportunity to possess great potential for use as an oligomeric network for drug reservoirs in in vitro application.

## 1. Introduction

Since 1960, when Wichterle and Lim published pioneering work on hydrogels (HGs) [1], interest in this group of hydrophilic polymers/oligomers has continued to grow, mainly in widely understood biomedicine, pharmacy, and bioengineering [2,3,4,5,6,7,8,9,10]. Properties such as the hydrophilic nature of the three-dimensional (3D) network, the ability to expand its volume by 10–20% after water absorption, and biocompatibility make these polymeric materials successfully used as matrices in tissue engineering [11,12,13,14], transdermal therapy [15], angiogenesis [16], antifungal therapy [17], chemotherapy [18], drug delivery [3,5,6,19], etc. Additionally, a fairly easy formation of the polymeric/oligomeric chains into macro-, micro-, and nano-sized gels gives unlimited possibilities for their versatile use, both in in vitro and in vivo studies [20,21].

Due to the origin and properties of HGs, they can be classified using different criteria [8,22], for example, (i) those obtained by the chemical synthesis and those of natural origin [4,23,24,25]; (ii) those chemically stable and those sensitive to external factors (thermo-responsive, electro-responsive, pH-sensitive, stimuli-responsive, etc.) [3,19,26,27,28]; (iii) those neutral and those with domains of different ionic characters [29,30,31], etc. The possibility of the controlled chemical and physical modification of polymeric chains gives the opportunity to design the 3D HGs matrix with targeted physico-chemical and biological properties [14], which consequently enable the design of a biomaterial with a specific application [8,9,14].

Polymers containing methacrylate units in their chains have been very well known for many years [32,33,34,35,36]. However, synthetic methacryloyl polymers can be precisely controlled and tailored to achieve desired properties, typically they show high hydrophobic aggregation, a lack of bioactivity, and low biodegradability. These properties significantly limit their application in broadly understood biomedicine and pharmaceutical sciences. On the other hand, the ease of combining various methacrylate derivatives into one polymer chain, in the ‘one-pot’ reaction, determines that the interest in these polymers is not waning [37,38,39,40,41]. Additionally, the modification of the polymeric methacryloyl chain is relatively easy and desirable [42]. The functionalization of the polymer chain alters its chemical and physical properties, which consequently allows the formation of HGs through chemical or physical forces. Chemically linked HGs use their ability to swell resulting from interactions among the polymer network, water, and the density of connections between polymer chains [43]. Physical HGs have chains connected by weak hydrogen bonds, ionic bonds and dipolar or hydrophobic interactions that form agar or gelatin [44,45].

The literature indicates that methacrylate-based polymers can be successfully used to create HGs, which can be further used in biomedical applications, for example to encapsulate cells, or enhance cell viability and neural differentiation [46,47]. For example, photopatterning was used for the preparation of microdevices with immobilized methacryloyl HGs [37,48,49]. Controlling the microarchitecture of the HGs results in an increase in their chemical and mechanical stability with higher biodegradability [48]. These results suggest that methacryloyl gelatin could be useful for cell-responsive microengineered HGs. The 3D HGs containing human-blood-derived endothelial colony-forming cells and bone-marrow-derived mesenchymal stem cells generate capillary-like networks in vitro that modulate the cellular behavior and the extent of vascular network formation both in vitro and in vivo [48]. Increasing the stiffness and rheological properties of gelatin methacryloyl HGs was also performed using its modified chain with glutamine and lysine groups, that were enzymatically crosslinked with a microbial transglutaminase [50]. Methacrylate gelatin HG was also successfully prepared using carboxybetaine methacrylate as a dopant, which may tune the mechanical properties of methacryloyl HG, its slower degradation rate, and a controlled drug release rate [38]. The results indicate that methacrylate gelatin/carboxybetaine methacrylate HGs are biocompatible and they are promising materials for drug delivery and tissue engineering. Combining poly(ethylene glycol) and methacrylated gelatin into HG resulted in the tuning of mechanical and degradation properties as well as in increasing fibroblast surface binding and spreading [51]. The doping of Si ions into gelatin methacrylate HG enhanced cellular adhesion and proliferation, therefore leading to the increased secretion of collagen I [47]. This study showed that Si-doped HGs were able to enhance the activity of human dermal fibroblasts due to the gradual release of Si ions. The Si-doped HG is a potential candidate for future skin regeneration clinical applications. Further modification of glycidyl methacrylate-based polymers with arginine-based poly(ester urea urethane) and chitosan resulted in the creation of cationic biodegradable HGs, that demonstrated excellent antibacterial activity [52]. The hybrid HGs exhibit great potential as antibacterial wound-dressing candidates for wound healing. These selected examples clearly indicate that methacryloyl HGs show great promise as tunable, cell-responsive HGs for 3D cell culture and regenerative medicine applications.

Here, chemical procedures for the formation of HGs containing ethyl methacrylate (EMA) and glycidyl methacrylate (GMA) monomers are presented. The oligomerization reaction leads to the formation of a random co-oligomer: poly[(ethyl methacrylate)-*co*-(glycidyl methacrylate)], [P(EMA)-*co*-(GMA)]. Their covalent functionalization with hydrophilic moieties leads to modification of the oligomeric chain, which makes it possible in the final stage to convert this hydrophobic oligomer into a 3D network of HGs. Additionally, the physico-chemical properties of the non-modified and the modified [P(EMA)-*co*-(GMA)] materials, the possibility of using them as a matrix for immobilizing model compound (drugs) and their cytotoxicity are presented.

## 2. Results and Discussion

### 2.1. HGs Preparation: Synthesis of [P(EMA)-co-(GMA)] and Their Covalent Functionalization with Hydrophilic Moieties

**Scheme 1:**
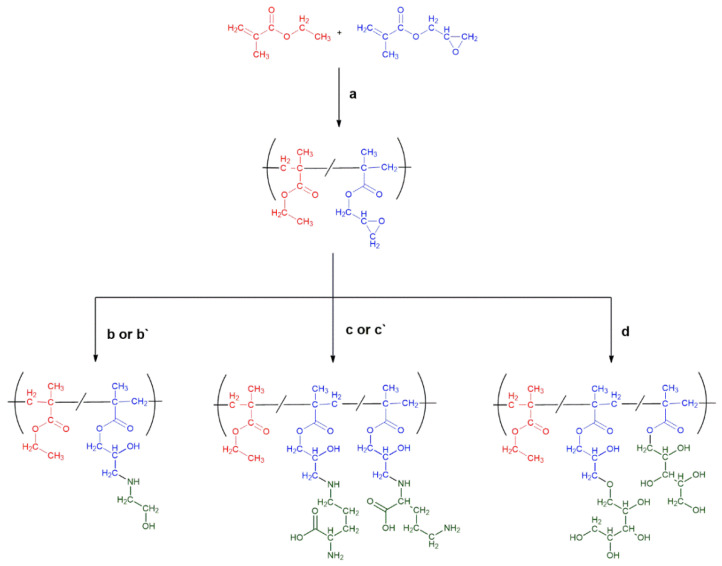
Presentation of the synthesis pathways of (**a**) the [P(EMA)-*co*-(GMA)] preparation (AIBN, heptane, 75 °C) and the covalent modification of the [P(EMA)-*co*-(GMA)] with (**b**) ETA (120 °C, 0.5 h); (**b`**) ETA (DMF, 120 °C, 0.5 h); (**c**) Orn (DMF/H_2_O, *v*/*v*, 8/2), 150 °C, 0.5 h); (**c`**) Orn (DMSO/H_2_O, *v*/*v*, 8/2), 150 °C, 0.5 h); (**d**) Xyl (DBU, DMF, 110 °C, 0.75 h).

To obtain the desired HGs, a suitable oligomer, consisting of two components, EMA and GMA, [P(EMA)-*co*-(GMA)], was prepared. Then, the oligomeric chain was modified with ethanolamine (ETA), xylitol (Xyl), or L-ornithine (Orn) to obtain the hydrophilic 3D co-oligomers. Presentation of the synthesis pathways of the [P(EMA)-*co*-(GMA)] is shown in Scheme 1. A detailed description of syntheses for the non-modified and the modified [P(EMA)-*co*-(GMA)] are included in the Section 3.

The standard method for the preparation of methacrylic acid ester oligomers/polymers is based on a radical polymerization reaction (initiated with AIBN), usually carried out in toluene solution, followed by precipitation of the resulting oligomer or polymer with methanol and further purification of the product [53,54]. Herein, the reaction was performed in heptane solution, which reduces the dispersion of the obtained product due to the dependence between the length of the oligomeric chain and its solubility in the reaction medium (Scheme 1) [55]. The longer chain of the oligomer reduced the solubility of the oligomer in the reaction medium. This process made it possible to obtain an oligomer chain using precipitation polymerization. The experimentally selected molar ratio of EMA to GMA and to initiator (AIBN) was 1:2.6:0.2 (EMA:GMA:AIBN, respectively). Based on the integration of signals of the characteristic protons in the ^1^H NMR spectrum of [P(EMA)-*co*-(GMA)] (Figure S1, Supplementary Materials), the ratio of EMA to GMA mers was 1:2.5. Characteristic signals 2H (Figure S1, signal ‘g’) at 3.99 ppm for EMA and 1H (Figure S1, signal ‘e’) at 3.19 ppm for GMA show an integration of 0.41 and 1.00, respectively. A detailed description of ^1^H NMR and ^13^C NMR spectra of the non-modified and the modified [P(EMA)-*co*-(GMA)] oligomers is presented in the Materials and Methods. NMR spectra for the remaining oligomers are presented in Supporting Information (Figures S1–S6).

Furthermore, the functionalization of [P(EMA)-*co*-(GMA)] was performed. We have developed a method of post-modification of [P(EMA)-*co*-(GMA)] using microwave-assisted synthesis. The procedure was based on the covalent reaction between [P(EMA)-*co*-(GMA)] oligomer and reactant (ETA, Xyl or Orn) dissolved in the different medium. Research on the synthesis and modification of the P(GMA) chains through reactions with various aza-nucleophiles allow us to state that they proceed mainly in the direction of the epoxide ring opening [56,57]. Moreover, the place of the attachment of aza-nucleophile is the least sterically shielded terminal CH_2_(O) epoxide carbon atom. It is also the strongest electrophilic centre. Studies on the regioselectivity of the nucleophilic epoxide opening reaction with the participation of ETA have been already described in the literature [58]. The reaction is mainly aimed at obtaining a product resulting from the attack of the amino group (aza-nucleophile) on one of the electrophilic centres of the epoxide group (Scheme 1).

Taking into account the “strength” of the nucleophile, it is commonly known that the amino group is significantly more nucleophilic than the hydroxyl group [59]. However, the [P(EMA)-*co*-(GMA)] opening reaction with an alkoxy anion, resulting from the deprotonation of the -OH group, is also possible. Most likely, in the reaction mixture with the participation of the aforementioned anion, the epoxy group is also opened and both ester groups of the oligomer/polymer are trans-esterified. However, the share of the products of the above reactions is less significant. It is also known that the secondary amine formed by the reaction of ETA with the epoxy group of [P(EMA)-*co*-(GMA)] subsequently reacts with the epoxy group of the adjacent oligomer/polymer chain, leading to cross-linking and, consequently, obtaining the 3D structure of the oligomer/polymer. Depending on the amount of amine used, the degree of cross-linking may vary, and thus the properties of the HG may be controlled [60].

A similar cross-linking mechanism may take place in [P(EMA)-*co*-(GMA)] reaction with Orn. Modifications of P(GMA) using a homologue of this amino acid (L-lysine) are also well known in the literature [61]. It is postulated that the main reaction taking place with the participation of this type of polymer is the epoxy group opening reaction by the amino group of the amino acid. Orn, apart from the presence of two amino groups, also has a carboxyl group, which can also act as a nucleophile that leads to attack the electrophilic epoxide centres [62]. However, it has been underlined that [P(EMA)-*co*-(GMA)] may react with Orn in different ways. Taking into account the presence of two amino groups in the oligomeric chain, which are strong nucleophiles, and the advantage of the number of glycide groups in the oligomer over epoxy groups (Scheme 1, path (b) or (b`)), we have proposed the most likely reaction pathways, giving the [P(EMA)-*co*-(GMA)]-Orn product.

[P(EMA)-*co*-(GMA)]-Xyl was synthesized as a result of the reaction between [P(EMA)-*co*-(GMA)] and Xyl in anhydrous DMF using microwave-assisted synthesis (Scheme 1, path (d)). Due to the difference in acidity of primary and secondary alcohol, 1,8-diazabicyclo(5.4.0)undec-7-ene (DBU) was used as a catalyst for post-polymerization reaction, acting as a strong base to deprotonate the -OH group of Xyl [63]. Additionally, the steric availability of the -OH groups in the reaction with the participation of Xyl as a nucleophile was mainly based on the participation of the primary -OH groups. The utility of DBU in DMF as a catalyst for the nucleophilic opening of P(GMA) epoxy groups with polyhydroxy compounds (e.g., cyclodextrins) has already been described in the literature and successfully applied in post-modification of this polymer [64,65]. DBU is also a well-known trans-esterification catalyst. Due to stereoelectronic effects (high steric hindrance), in the vicinity of the carbonyl electrophilic centre of the ester groups, the trans-esterification reaction with Xyl should preferably follow the aforementioned mechanism of nucleophilic opening of the epoxide groups.

### 2.2. Physico-Chemical Characteristics of [P(EMA)-co-(GMA)] and Their Derivatives

**Figure 1:**
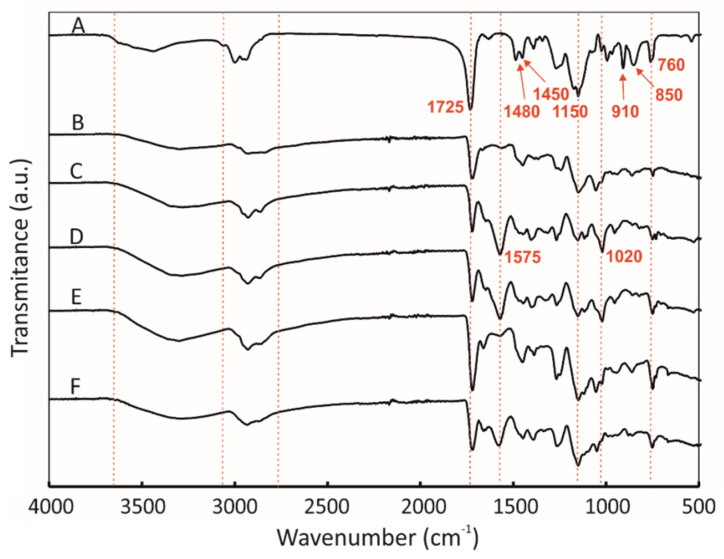
FT-IR spectra of (**A**) [P(EMA)-*co*-(GMA)], (**B**) [P(EMA)-*co*-(GMA)]-ETA(**1**), (**C**) [P(EMA)-*co*-(GMA)]-Orn(**1**), (**D**) [P(EMA)-*co*-(GMA)]-Xyl, (**E**) [P(EMA)-*co*-(GMA)]-ETA(**2**), and (**F**) [P(EMA)-*co*-(GMA)]-Orn(**2**).

Fourier transform infrared spectroscopic (FT-IR) method was used to analyse the chemical structures of the synthesized oligomers. Figure 1 shows the spectra of the non-modified and the modified [P(EMA)-*co*-(GMA)]. For the non-modified oligomeric networks (Figure 1A), two strong characteristic peaks were observed at about 1725 and 1150 cm^−1^, which are attributed to C=O and C-O stretching vibrations, respectively. The oligomers show the bands corresponding to the stretching vibrations of C-O at 910 cm^−1^ and C-O-C at 850 cm^−1^. The epoxy groups in the polymeric network gave an absorption band due to the vibration near 915 cm^−1^ (bending of the -C-O-C- triad) [66,67]. The extent of epoxy can be monitored by measuring the intensity of this band.

The characteristic vibrations of the aliphatic C-H groups could also be seen: at 3000 and 2930 cm^−1^ stretching vibrations C-H of aliphatic CH_2_, at 1448 cm^−1^ deformation vibrations C-H of CH_2_ and CH, and at 755 cm^−1^ rocking vibrations -CH_2_. Only those FT-IR bands which are essential for the formation of the oligomeric networks and their functionalization are described below. The broad intense band at about 3600–3000 cm^−1^ represents the stretching vibrations of H-bonded hydroxyl (O-H) groups of alcoholic groups in HGs (Figure 1B–F). This wide band may be also attributed to the overlapping of the N-H vibrations from the modified oligomeric network.

The weak C-H vibration bands that are observed for the modified [P(EMA)-*co*-(GMA)] at about 2930 cm^−1^ were superimposed as a shoulder and are assigned due to vibrations of asymmetric and symmetric aliphatic groups (CH_3_ and CH_2_). The infrared stretching frequency of the C≡N group of the initiator attached to the oligomeric chain is observed at about 2250 cm^−1^. The bands at the ranges of 1750–1720 cm^−1^ and 1150–1000 cm^−1^ can be assigned to the stretching vibrations of C=O groups and the stretching and the deformation vibrations of C-O (Figure 1C,D,F), respectively [68]. For the [P(EMA)-*co*-(GMA)] modified with ETA and Orn, we also observed bands at 1570 cm^−1^ (ETA, Figure 1B,E) and 1575 cm^−1^ (Orn, Figure 1C,F), which can be attributed to N-H bending vibrations. The weak C-N stretching bands at 1020 cm^−1^ are also observed for the [P(EMA)-*co*-(GMA)] derivatives.

In order to confirm the proposed synthesis pathways of the oligomers, the molar mass distribution and the average molar mass of the methacrylate oligomer and their derivatives were determined using the size-exclusion (gel) chromatography technique (HPLC-GPC/SEC). The results of the determination of the average molar mass for the methacrylate oligomer derivatives obtained with the use of size exclusion chromatography are presented in Table 1, while the molar mass distribution for the fractions tested are presented in Tables S1–S4.

The molar mass distribution for the oligomers and the dispersity index (*Ð*), with a value close to 1.1, indicate that the oligomers, which were synthesized, are homogeneous material with a fairly homogeneous distribution of the length of the oligomer chain. The molecular mass distribution for [P(EMA)-*co*-(GMA)] in the range from 601.2 to 300.8 Da represents 86.84% of the total oligomer mass with an average molar mass of 436.50 (Table 1 and Table S1). Similarly, [P(EMA)-*co*-(GMA)]-ETA(**2**) is a homogeneous oligomer where 88% is oligomer with a mass in the range of 601.3–217.1 Da and *Mn* of 473.20 (Table 1 and Table S3). The molecular mass distribution for [P(EMA)-*co*-(GMA)]-Xyl and [P(EMA)-*co*-(GMA)]-Orn(**2**) is more varied; about 94% of the total mass of the oligomer ranges between 1004.2 and 217.1 Da, with the highest value of *Ð* at approx. 1.17 (Table 1, Tables S2 and S4). Average molar mass values for the methacrylate oligomer derivatives calculated based on HPLC-GPC/SEC clearly indicate that the resulting chains are short and have about three or four units (mers) in the oligomer.

Based on the results obtained by size-exclusion chromatography and ^1^H NMR, the main conclusions can be made as follows. First, for [P(EMA)-*co*-(GMA)], the number of EMA to GMA units is ca. 1:2. Second, the [P(EMA)-*co*-(GMA)] oligomer is incompletely functionalized with ETA. The presence of two singlets at 2.67 and 2.87 ppm in the ^1^H NMR spectrum [P(EMA)-*co*-(GMA)]-ETA(**1**) proved the incomplete conversion of the epoxy groups into ETA-functionalized [P(EMA)-*co*-(GMA)]. The number of EMA to GMA units in [P(EMA)-*co*-(GMA)]-ETA is approximately 1:2.5. Next, the reaction of [P(EMA)-*co*-(GMA)] with Xyl under the influence of DBU, used as a catalyst, gives a compound with a mass of 713.77 g mol^−1^. The functionalization reaction takes place with the opening of the epoxide group, as well as the trans-esterification of the glycide group.

In order to confirm the structure of the synthesized oligomer chains, we also performed the titration method (also known as the hydrochloric acid-acetone-standing method) of the epoxy groups for the starting oligomer [P(EMA)-*co*-(GMA)]. The number of epoxy groups (*L_ep_*) was calculated [69] using the formula below:

(1) $$L_{ep}=\frac{\left(V_{0}-V_{1}\right)\times k_{1}\times 43}{m\times 10}\;\%$$

where *V*_0_ is the volume of titrant used per standard (blank sample) in mL, *V*_1_ is the volume of titrant used per oligomer in mL, *k*_1_ is a factor of 1 for a 0.1 M NaOH standard solution, 43 is the mass of epoxy group in g mol^−1^ and *m* is the sample mass in g. The obtained results are summarized in Table S5. The ratio of the number of EMA to GMA units was estimated and it is 0.21 to 1, which in combination with the results obtained by HPLC-GPC/SEC and ^1^H NMR methods, allows us to conclude that the EMA to GMA ratio in [P(EMA)-*co*-(GMA)] is 1:2.5. Summarizing, all three methods confirmed the proposed structure of the oligomeric chain [P(EMA)_x_-*co*-(GMA)_y_], which can be represented as the number of units of x to y as 1:2.5, respectively.

**Figure 2:**
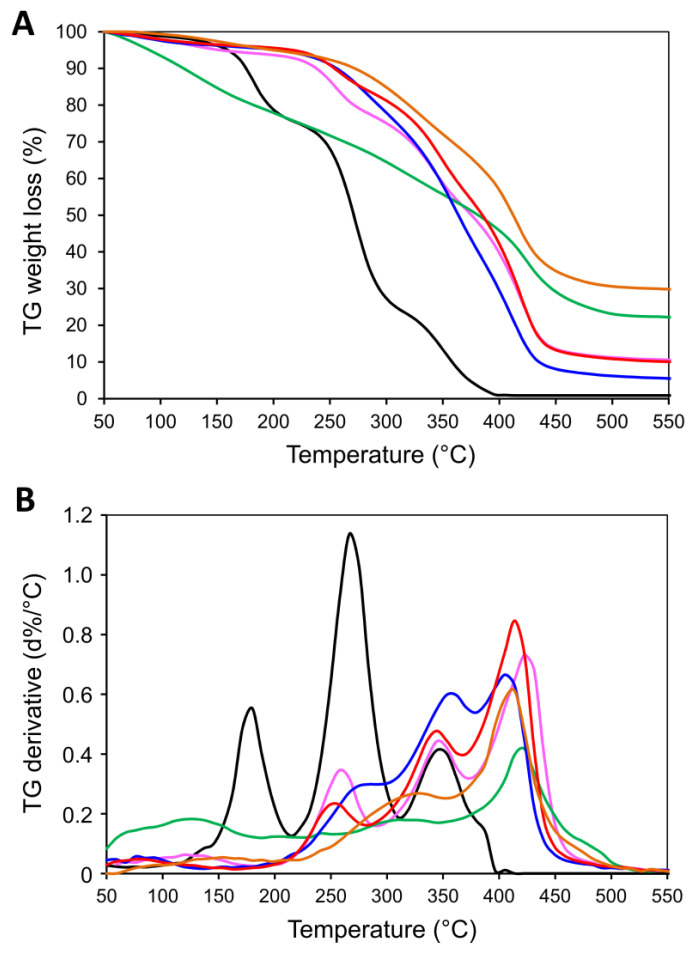
TGA (**A**) and DTG (**B**) of [P(EMA)-*co*-(GMA)] (black) [P(EMA)-*co*-(GMA)]-ETA(**1**) (purple), [P(EMA)-*co*-(GMA)]-Orn(**1**) (green), [P(EMA)-*co*-(GMA)]-Xyl (blue), [P(EMA)-*co*-(GMA)]-ETA(**2**) (red), and [P(EMA)-*co*-(GMA)]-Orn(**2**) (orange). Measurements were performed in an Ar atmosphere at a rate of 10 °C min^−1^.

Differential thermogravimetric measurements (TGA-DTG) were performed to analyse the thermal stability of the non-modified and the modified [P(EMA)-*co*-(GMA)] (Figure 2). Figure 2A,B show the TGA-DTG curves recorded up to 550 °C in an Ar atmosphere at rate 10 °C min^−1^. TGA measurements were performed at the ambient temperature. Samples undergo thermal decomposition in the range of 150 to 500 °C. TGA-DTG curves of [P(EMA)-*co*-(GMA)] have several degradation steps: 150–220 °C, 220–320 °C (the main mass lost), and 320–400 °C (Figure 2A), with the maximum of the degradation at 180, 267 and 353 °C, respectively (Figure 2B).

Generally, the products formed during the degradation of [P(EMA)-*co*-(GMA)] are monomers (mainly P(EMA) and P(GMA)), and byproducts—the result of ester decomposition (e.g., allyl alcohol, acrolein, glycidol, propene, isobutene, CO_2_, and CO) [70,71]. The mass losses recorded at the temperature range of 150–500 °C for oligomeric derivatives are between 70 and 95%. They depend on the reaction conditions and used modifiers (ETA, Orn or Xyl).

Almost all post-modified oligomers show higher thermal stability than the starting materials, which confirms the modification of the epoxy groups with ETA, Orn or Xyl. Only [P(EMA)-*co*-(GMA)]-Orn(**1**) has almost linear decomposition in the range of 50–450 °C. On the other hand, the most stable was the same product obtained in a slightly different experimental conditions [P(EMA)-*co*-(GMA)]-Orn(**2**). Its decomposition started above 250 °C and occurred in two main steps at the temperature range of 250–350 °C and 350–450 °C (Figure 2A) with the maximum at the temperatures of 330 and 450 °C, respectively (Figure 2B). The observed residuals, at the level of approximately 30%, are probably related to the carbonization process, which occurs when the samples are isolated from the oxygen during measurement. These results clearly show that the thermal stability of the synthesized oligomers is related to the chemical structures of their chains, and the method used for their functionalization with hydrophilic moieties.

**Scheme 2:**
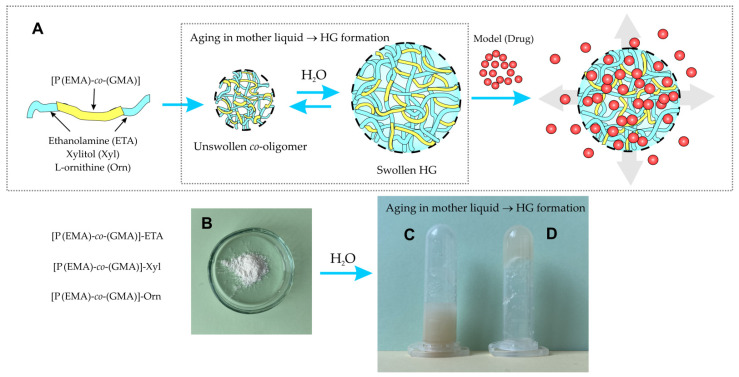
(**A**) Graphical representation of HGs preparation. Photographs of (**B**) *co*-oligomer, (**C**) *co*-oligomer with H_2_O, and (**D**) *co*-oligomer after aging (HG).

Synthesis of the [P(EMA)-*co*-(GMA)] and their further modification with moieties containing –OH and –NH_2_ groups (Xyl, ETA or Orn) allow us to obtain the products in the form of white or slightly yellow solid powders (Scheme 2). The addition of water to the [P(EMA)-*co*-(GMA)] enabled the formation of HGs. The key to the successful preparation of the modified [P(EMA)-*co*-(GMA)] is a judicious selection of monomers and a hydrophilic component. The optimal conditions of the procedure established in our group for these HGs’ preparation are presented, mainly taking into account the structural porosity and water uptake of the final materials. The schematic preparation of HGs from the modified [P(EMA)-*co*-(GMA)] is shown in Scheme 2. The resulting HGs are stable over time, but upon removal of the solvent from their networks, they reform a solid powder.

**Figure 3:**
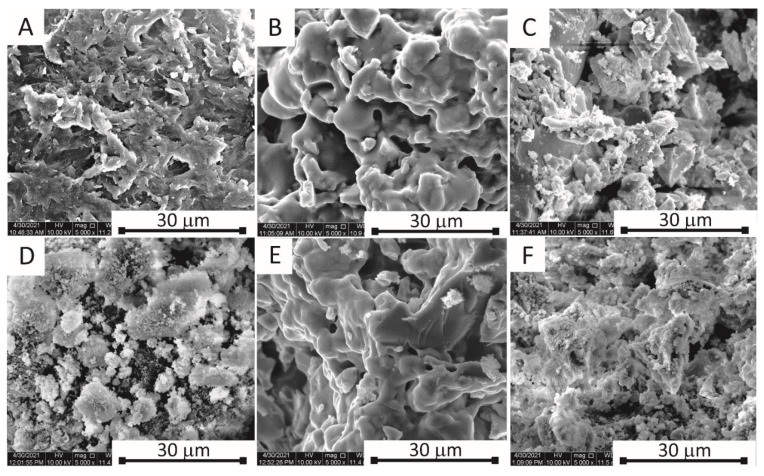
SEM images of (**A**) [P(EMA)-*co*-(GMA)], (**B**) [P(EMA)-*co*-(GMA)]-ETA(**1**), (**C**) [P(EMA)-*co*-(GMA)]-Orn(**1**), (**D**) [P(EMA)-*co*-(GMA)]-Xyl, (**E**) [P(EMA)-*co*-(GMA)]-ETA(**2**), and (**F**) [P(EMA)-*co*-(GMA)]-Orn(**2**).

Microscopic characterization was performed to evaluate the morphology of dried films prepared from the synthesized oligomers. Scanning electron microscope (SEM) images of films of the non-modified and the modified [P(EMA)-*co*-(GMA)] are shown in Figure 3. Films were prepared by drop-coating of the individual oligomers dispersed in an ethanol solution and solvent evaporation. Next, the surface of the oligomeric films was covered with a Au layer of (7 nm thickness) to minimize the electrifying effect of obtained film in contact with the electron beam.

The morphology of the non-modified [P(EMA)-*co*-(GMA)] differs from the oligomers modified with ETA, Xyl, and Orn. At the micrometer scale, the non-modified [P(EMA)-*co*-(GMA)] formed aggregates in an ethanol solution, showing petal-like morphology (Figure 3A). The modification of [P(EMA)-*co*-(GMA)] with hydrophilic moieties increased their dispersibility in protic solvents, which is shown in Figure 3C,D,F. Only the functionalization of [P(EMA)-*co*-(GMA)] with ETA leads to a reduction in the porosity of the obtained oligomer films, and their surface morphology appears more homogeneous (Figure 3B,E).

A characteristic feature of HGs is their ability to uptake water, known also as swelling behaviour. To evaluate the water uptake of the synthesized oligomers, the following procedure was performed. Of each dry sample, 1 g was weighed and placed in a glass container. Distilled water was poured into the vessel and it was left to swell for 1 h. After this time, the sample was filtered on a paper filter and the residue was weighed. The results were collected and the swelling degree (*SW*) was calculated using the following formula [72]:

(2) $$SW=\frac{m_{swollen}-m_{dried}}{m_{dried}}$$

where *m_dried_* is the mass of the dried substance before swelling (water uptake), and *m_swollen_* is the mass of the oligomer after swelling (water uptake) in equilibrium. The results of the experiment on the water uptake of synthesized materials are summarized in Table 2.

As can be seen from the results collected in Table 2, the [P(EMA)-*co*-(GMA)]-ETA(**2**) oligomer has the best swelling properties with a value of 4.15. Compared with [P(EMA)-*co*-(GMA)]-Orn(**1**), which has the lowest *SW* (1.25), this may be due to insufficiently drained water. The test tube inversion showed that [P(EMA)-*co*-(GMA)]-ETA(**1**), [P(EMA)-*co*-(GMA)]-ETA(**2**) and [P(EMA)-*co*-(GMA)]-Orn(**2**) gel very well. [P(EMA)-*co*-(GMA)]-Orn(**1**), which did not sufficiently gel to remain in the tube, nevertheless forms a dense structure that can be considered as a gel.

### 2.3. Release of Model Compounds from HGs Network

For our applications, where the absorption of water (swelling behaviour) is not the most crucial, each of the oligomers can be used for further studies. Due to the fact that the HG used as a matrix for immobilizing the active substances should have a “compact structure” of the polymer chain, where the polymer material must have adequate mechanical stability and stiffness, a small amount of 10% agarose was added for the release of model compounds from the HGs matrix. The study was carried out at 37 °C, simulating the average body temperature.

**Figure 4:**
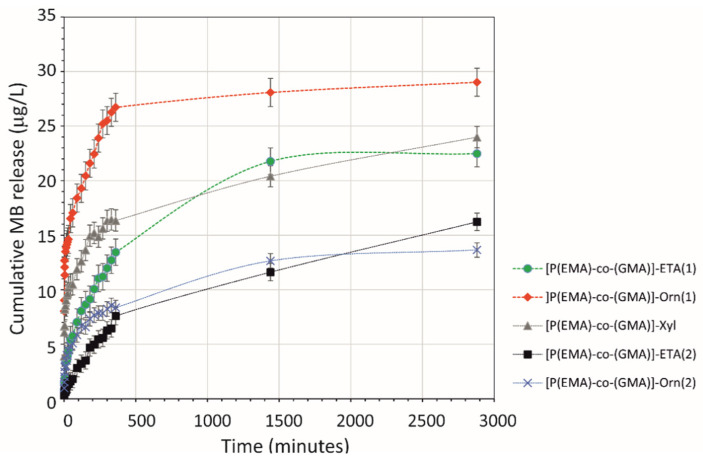
Release profiles of MB from the synthesized oligomers. Determination of the MB concentration was based on spectrophotometric analysis at λ = 633 nm.

Methylene blue (MB) was used as a model compound to study the release from the gel matrix, due to its hydrophilic properties and easy determination by UV-vis spectrophotometric methods. The release of MB from the HGs matrix was carried out for all modified oligomers (Figure 4). Briefly, drug release was performed according to the following procedure. Of each oligomer, 100 mg was mixed with 2 mg of MB and 500 μL of 10% agarose solution in PBS (pH = 7.4). After approximately 24 h, when the HG had formed in a tube with a strictly defined diameter, the tube was immersed with one end in a beaker with 30 mL of PBS solution (pH = 7.4) at 37 °C with stirring. At defined intervals, a sample of 100 μL was taken and the spectrophotometric determination of MB was performed. A detailed description of the experimental parameters is included in Section 3.

The results show that the release of MB loaded into the HG is highest and fastest during the first 6 h (Figure 4). After this time, the release is reduced until it reaches equilibrium with the solution, which was observed during the 48 h of the experiment. The obtained results indicate that the obtained oligomers can be used in the future to develop materials for controlled, prolonged release of hydrophilic active substances.

### 2.4. Biological Studies

**Figure 5:**
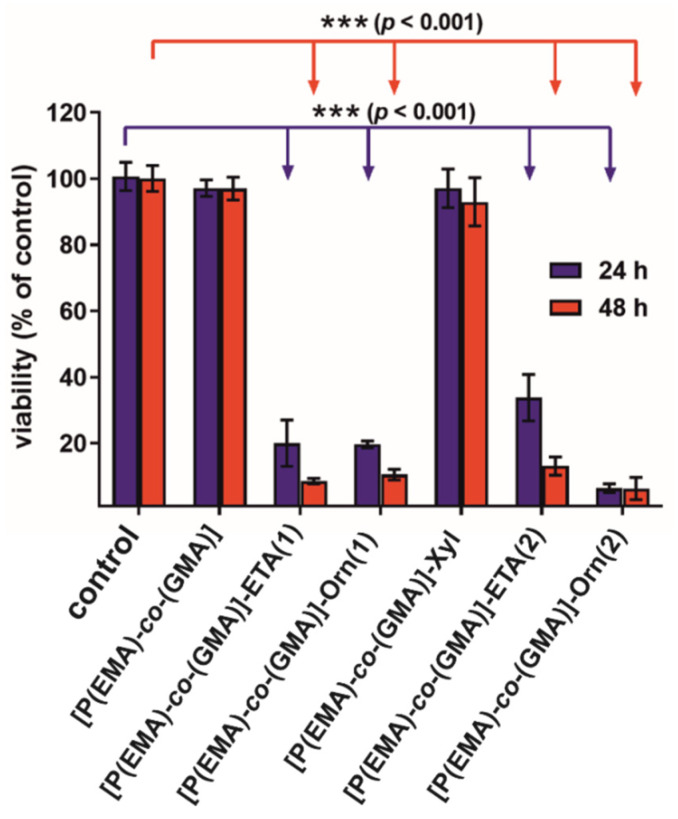
MTT cytotoxicity test performed according to ISO 10993-5 standard using fluid extracts of HGs and human skin fibroblasts (*** statistically significant results compared to the control, according to one-way ANOVA followed by Dunnett’s test).

The cytotoxicity test showed the non-toxicity of only non-modified [P(EMA)-*co*-(GMA)] and [P(EMA)-*co*-(GMA)]-Xyl (Figure 5). Cells exposed to extracts of the mentioned oligomers exhibited high viability that was near 100% throughout the full length of the experiment. Other samples were characterized by high cytotoxicity since cell viability was significantly reduced to approx. 20% for [P(EMA)-*co*-(GMA)]-ETA(**1**) and [P(EMA)-*co*-(GMA)]-Orn(**1**), to 35% for [P(EMA)-*co*-(GMA)]-ETA(**2**) and to 7% for [P(EMA)-*co*-(GMA)]-Orn(**2**). Moreover, the extension of exposure time to 48 h resulted in a further reduction in cell viability.

Based on the cell culture experiment it may be concluded that only [P(EMA)-*co*-(GMA)] and [P(EMA)-*co*-(GMA)]-Xyl have the potential to be used in biomedical applications as drug carriers. Unfortunately, the first of them, due to its hydrophobic nature, cannot be used to prepare the HG matrix in its non-modified form. On the other hand, its non-toxicity indicates the possibility of using [P(EMA)-*co*-(GMA)] as a template for further functionalization.

## 3. Materials and Methods

### 3.1. Materials

All solvents (dimethylformamide (DMF), ethyl aceteate (AcOEt), hexane, heptane, toluene, methanol (MeOH), ethanol (EtOH), isopropyl alcohol (IPA)) were purchased from POCH (Gliwice, Poland) and distilled prior to use. DMF was dried by vacuum distillation over P_2_O_5_ under the N_2_ atmosphere. Heptane and toluene were distilled in the same way but under normal pressure. The remaining reagents were purchased from SigmaAldrich (Poznań, Poland) (glycidyl methacrylate (GMA, ≥97% GC), ethyl methacrylate (EMA, ≥99% GC), ethanolamine (ETA, ≥99%), l-ornithine hydrochloride (Orn), xylitol (Xyl), azobisisobutyronitrile (AIBN, ≥95%), 1,8-diazabicyclo(5.4.0)undec-7-ene (DBU), sodium hydroxide (NaOH), hydrochloric acid (HCl), agarose, phosphate-buffered saline (PBS, pH = 7.4), methylene blue (MB) and were of analytical grade, and used without prior purification. Before using methacrylic esters for polymerization, these monomers were filtered through a short layer of dry silica gel (40–60 mm) to remove polymerization inhibitors.

#### 3.1.1. Synthesis of Poly(ethyl methacrylate)-*co*-poly(glycidyl methacrylate) [P(EMA)-*co*-(GMA)]

The polymerization reaction was carried out under an atmosphere of nitrogen and anhydrous conditions. EMA (5.76 g, 50.3 mmol), GMA (18.70 g, 131.6 mmol), solid AIBN (2.0 g, 12.2 mmol), and 200 mL anhydrous heptane were added to a 1 L round bottom flask equipped with a heating and magnetic stirrer. The obtained solution was then heated at 75 °C for 3 h. During this time, the product precipitated from the solution in the form of a powder. After the set time, the reaction flask was cooled to room temperature. The reaction mixture was filtered under vacuum in a Büchner funnel, and the precipitate was washed with a small amount of AcOEt/hexane (2/98, *v*/*v*) solution. Finally, the crude product was transferred to a round bottom flask and volatile residues were removed on a rotary evaporator under reduced pressure (20 °C, 50 mbar). [P(EMA)-*co*-(GMA)] was obtained in the form of a white solid (21.80 g), well soluble in THF, MeCN, acetone, DCM, and AcOEt, and slightly less in EtOH. ^1^H NMR (400 MHz, DMSO-*d*_6_), δ (ppm): 0.82 and 0.99 (br, backbone CH_3_), 1.21 (br, ethyl ester CH_3_), 1.83–1.89 (m, backbone CH_2_), 2.67–2.81 (m, epoxide CH_2_), 3.21 (br, epoxide CH), 3.74 (br, glicydyl ester-CH_2_-epoxide), 3.99 (br, ethyl ester-CH_2_), 4.31 (br, glicydyl ester-CH_2_-epoxide). ^13^C NMR (176 MHz, DMSO-*d*_6_), δ (ppm): 176.9 and 175.8 (>C=O), 79.2 and 65.7 (>C-OR), 60.5, 53.5, 48.6, 48.5, 18.4, 16.5, 13.5.

#### 3.1.2. Synthesis of [P(EMA)-*co*-(GMA)] with ETA {[P(EMA)-*co*-(GMA)]-ETA(**1**)}

[P(EMA)-*co*-(GMA)] (0.50 g) and ethanolamine (ETA) (5.01 g, 81.9 mmol) were added to a 30 mL tube. A stream of N_2_ was bubbled through the mixture and the tube was sealed with an adapter. The system was heated by microwave (120 °C, 30 min). After the set time, the reaction mixture was transferred to a centrifuge tube with AcOEt/acetone (1/1, *v*/*v*) to a volume of 50 mL. The tube was sealed and the resulting gelatinous suspension was shaken vigorously by hand for one minute, and the pellet was centrifuged in a rotary centrifuge (10 min, 7000 rpm). The decanted solution was discarded and the washing of the precipitate was repeated two more times with fresh portions of the AcOEt/acetone mixture (1/1, *v*/*v*). Finally, the crude product was dried on a rotary evaporator under reduced pressure (90 °C, 50 mbar) to give [P(EMA)-*co*-(GMA)]-ETA(**1**) as a white powder (0.51 g). ^1^H NMR (400 MHz, DMSO-*d*_6_), δ (ppm): 0.76 and 0.92 (br, backbone CH_3_), 1.17 (br, ethyl ester CH_3_), 1.77–1.89 (m, backbone CH_2_), 2.59 (br, -CH_2_-NH-CH_2_-), 3.34–3.37 (m, O-CH_2_-CH<), 3.76 (br, CH_2_-OH), 3.96 (br, CH_2_ in Et).

#### 3.1.3. Synthesis of [P(EMA)-*co*-(GMA)] with ETA in DMF {[P(EMA)-*co*-(GMA)]-ETA(**2**)}

[P(EMA)-*co*-(GMA)] (0.50 g), ETA (1.02 g, 16.7 mmol) and anhydrous DMF (10 mL) were added to a 30 mL tube. A stream of N_2_ was bubbled through the mixture and the tube was sealed with an adapter. The system was heated by microwave (120 °C, 30 min). After the set time, the reaction mixture was transferred to a centrifuge tube with AcOEt/hexane (8/2, *v*/*v*, total volume 80 mL) solution. The resulting suspension was cooled to 0 °C and was centrifuged during 10 min (7000 rpm). After decanting the solution, a mixture of AcOEt/acetone (1/1, *v*/*v*, total volume 50 mL) was added to the solid residue. The resulting mixture was homogenized with a glass rod and then shaken by hand in a capped vial for one minute. The pellet was centrifuged and the solution, after previous decanting, was discarded. The washing of the precipitate was repeated additionally with one more portion of the AcOEt/acetone (1/1, *v*/*v*) solution. Finally, the crude product was dried on a rotary evaporator under reduced pressure (90 °C, 50 mbar) to give [P(EMA)-*co*-(GMA)]-ETA(**2**) as a white powder (0.49 g). If the -synthesized product did not have a powdery texture after drying, it was powdered in a mortar with a small amount of diethyl ether, followed by removal of the solvent under reduced pressure. ^1^H NMR (400 MHz, DMSO-*d*_6_), δ (ppm): 0.76 and 0.92 (br, backbone CH_3_), 1.12–1.17 (m, ethyl ester CH_3_), 1.77–1.89 (m, backbone CH_2_), 2.59 (br, -CH_2_-NH-CH_2_-), 3.34–3.37 (m, O-CH_2_-CH<), 3.75 (br, CH_2_-OH), 3.97 (br, CH_2_ in Et).

#### 3.1.4. Synthesis of [P(EMA)-*co*-(GMA)] with Xyl {[P(EMA)-*co*-(GMA)]-Xyl}

[P(EMA)-*co*-(GMA)] (0.50 g), well powdered Xyl (0.50, 3.3 mmol), DBU (0.07 g, 0.5 mmol) and anhydrous DMF (10 mL) were added to a 30 mL tube. A stream of N_2_ was bubbled through the mixture and the tube was sealed with an adapter. The system was heated by microwave (110 °C, 45 min). After the set time, the reaction mixture was transferred to a centrifuge tube with AcOEt to a volume of 50 mL. The tube was sealed and the resulting suspension was shaken vigorously by hand for one minute and the pellet was centrifuged in a rotary centrifuge (10 min, 7000 rpm). The product was washed with 95% EtOH (2 × 50 mL). Finally, the crude product was dried on a rotary evaporator under reduced pressure (80 °C, 50 mbar) to give [P(EMA)-*co*-(GMA)]-Xyl as a white powder (0.52 g).

#### 3.1.5. Synthesis of [P(EMA)-*co*-(GMA)] with Orn {[P(EMA)-*co*-(GMA)]-Orn(**1**) and [P(EMA)-*co*-(GMA)]-Orn(**2**)}

[P(EMA)-*co*-(GMA)] (0.50 g), Orn (0.50, 3.0 mmol), NaOH (0.24 g, 0.6 mmol) and DMF/MeOH (8/4, *v*/*v*, total volume 10 mL), product assigned as [P(EMA)-*co*-(GMA)]-Orn(**2**); or DMSO/H_2_O (8/2, *v*/*v*, total volume 10 mL), product assigned as [P(EMA)-*co*-(GMA)]-Orn(**1**), were added to a 30 mL tube. The system was heated by microwave (150 °C, 30 min). After the set time, the reaction mixture was transferred to a centrifuge tube with AcOEt to a volume of 50 mL. The tube was sealed and the resulting suspension was shaken vigorously by hand for one minute, and the pellet was centrifuged in a rotary centrifuge (10 min, 7000 rpm). The product was washed with 95% EtOH (2 × 50 mL). Finally, the crude product was dried on a rotary evaporator under reduced pressure (80 °C, 50 mbar) to give [P(EMA)-*co*-(GMA)]-Orn in the form of a slightly yellow powder (c.a. 0.50 g).

### 3.2. Methods

Microwave-assisted synthesis was performed in Anton Paar Monowave 450 microwave reactor (Ashland, VA, USA) in 30 mL sealed tubes. The films were imaged by secondary electron scanning electron microscopy (SEM) using an FEI Tecnai S-3000N instrument (Tokyo, Japan). The accelerating voltage of the electron beam was 10 keV. To minimize the electrifying effect of the polymer surface in contact with the electron beam, the surface of the polymer material was coated with an Au layer of 7 nm thickness using the sputtering method.

The size exclusion (gel) chromatography technique (HPLC-GPC/SEC) was used: two columns connected in series, the first type Phenogel Linear, 5 μm, 300 × 7.8 mm (MERCK, Darmstadt, Germany) and the second type PLgel 5 μm, 100 Å, 300 × 7.8 mm (Agilent, Santa Clara, CA, USA). Tetrahydrofuran (THF) was used as an eluent. The study used a LaChrom Merck-Hitachi gradient liquid chromatograph (Burladingen, Germany) equipped with: a three-channel low-pressure gradient system, L-6200 pump, Rheodyne Rh-7725i dosing valve with a 20 μL dosing loop, thermostat, 2300 refractometric detector and UV-VIS/DAD 7450A, with HSM software (Entrust Deutschland GmbH, Düsseldorf, Germany); additionally, a six-way two-position valve V7226, to reverse the flow of the mobile phase in the column (backflash). To calibrate the molecular weight distribution, a solution of a mixture of narrowly dispersive polystyrene molecular weight standards, manufactured by Merck (Darmstadt, Germany), with a dispersity of each standard lower than 1.25 and with average molecular weights: 1,000,000; 100,000; 10,000; 500 and 58 Da was used. Test samples were dissolved in DMSO. After dissolving the samples, the solvent was evaporated and the methacrylate oligomer derivatives were dissolved in THF. The HPLC-GPC/SEC separation process was carried out at 33 °C. The mobile-phase volume flow rate was 1.0 mL min^−1^ and the dosing volume was 10 mL.

The ^1^H NMR spectra were recorded using an Agilent VNMRS system (Agilent, Santa Clara, CA, USA) operated at 400 MHz for ^1^H NMR and 101 MHz for ^13^C NMR spectra. Samples were prepared as solutions in *d*_6_-DMSO and chemical shifts were determined relative to the tetramethylsilane (TMS). The Fourier transform infrared spectroscopy (FT-IR) spectra were recorded in the range between 4000 and 400 cm^−1^ with a NICOLET IN10 MX infrared microscope (Thermo Fisher Scientific, Waltham, MA, USA) operated at room temperature under an N_2_ atmosphere. The spectra were collected with a resolution of 4 cm^−1^, 256 scans were averaged to obtain a single spectrum and apodized with a triangular function, and a zero-filling factor of 1 was applied. All the spectra were corrected with conventional software to eliminate the variation in the analysed thickness with the wavelength.

Thermogravimetric analyses (TGA) were performed on a Mettler Toledo Star1 TGA/DSC unit (Mettler-Toledo Sp. z o.o., Warsaw, Poland). Samples weighing 2–3 mg were placed in aluminium oxide crucibles and heated from 50 to 900 °C at a heating rate of 10 °C min^−1^ under an Ar flow rate of 20 mL min^−1^.

### 3.3. Determination of Epoxy Groups for [P(EMA)-co-(GMA)]

To evaluate the amount of epoxy group for [P(EMA)-*co*-(GMA)] the titration method was performed. The hydrochloric acid–acetone mixture solution (1:40, *v*/*v*, in mL, respectively) was prepared. As indicator, 0.1% of phenolphthalein was used at neutral pH. Into a conical flask with a ground glass stoper, a certain amount of oligomer was weighed (0.2 = 0.0001 g) (Table S5). Subsequently, 8 mL of HCl–acetone solution was added to the flask and stoppered. After 2 h, 3–5 drops of indicator was added and the solution was titrated with a NaOH standard solution (the colour did not fade for 30 s). A blank experiment, in which no oligomer was dissolved in the HCl–acetone solution, was performed in the same way. The experiments were repeated three times.

### 3.4. In Vitro Release Studies

The following method was used to assess the release of the model substances. Of each oligomer, 100 mg was mixed with 2 mg of MB and placed in a plastic tube (0.4 cm of the diameter). Then, 500 μL of 10% agarose solution in PBS (pH = 7.4) was added to the tube and mixed, after which the tube was placed in an ultrasonic bath for 30 min at 60 °C and then in the refrigerator overnight. After this time, the tube was immersed with one end in a beaker with 30 mL of PBS solution (pH = 7.4) at 37 °C with stirring (100 rpm). At defined intervals, a sample of 100 μL was taken and the deficiency was refilled with fresh PBS solution. Spectrophotometric analysis was performed on a spectrophotometer (Epoch™ 2 Microplate Spectrophotometer, Agilent, Santa Clara, CA, USA) at 663 nm.

### 3.5. Preparation of Oligomeric Extracts

The extracts were prepared according to ISO 10993-12 standard by immersing 100 mg of oligomeric sample in 1 mL of complete culture medium followed by 24 h incubation at 37 °C. The complete culture medium consisted of Eagle’s Minimum Essential Medium (EMEM, ATCC-LGC Standards, Teddington, United Kingdom) with the addition of 10% foetal bovine serum (FBS, Pan-Biotech GmbH, Aidenbach, Bavaria, Germany) and a mixture of antibiotics: 100 U mL^−1^ penicillin and 100 μg mL^−1^ streptomycin purchased from Sigma-Aldrich Chemicals (Warsaw, Poland). After incubation, the extracts were collected, filtered using syringe filters, and centrifuged for 40 min at 10,000 rpm.

### 3.6. Cytotoxicity Test

Cytotoxicity of the oligomers was assessed according to ISO 10993-5 standard using fluid extracts of the samples and human skin fibroblasts (BJ cell line CRL-2522^TM^ obtained from ATCC-LGC Standards, Teddington, United Kingdom). The cells were maintained in the complete culture medium using a cell culture incubator (37 °C, humidified atmosphere of 5% CO_2_ and 95% air). A total of 2 × 10^4^ of BJ cells were seeded into the wells of 96-multiwell plates in 100 µL of complete culture medium. After 24 h culture at 37 °C, the culture medium was removed and replaced with extracts of the oligomers. Control cells (negative control of cytotoxicity), considered to exhibit 100% viability, were cultured in the complete culture medium, which was incubated for 24 h at 37 °C in a polypropylene tube but without a HG sample. Fibroblasts were exposed to HG extracts for 24 h and 48 h and then their viability was determined by MTT assay (Sigma-Aldrich Chemicals, Warsaw, Poland) according to the procedure described earlier [72]. Cytotoxicity test was performed in quadruplicate in six independent experiments. Statistically significant results between the control group and tested samples were considered at *p* < 0.05 according to one-way ANOVA followed by Dunnett’s multiple comparison test (GraphPad Prism 8.0.0 Software, GraphPad Software Inc., California, CA, USA).

## 4. Conclusions

Here, the synthesis of the oligomeric network [P(EMA)-*co*-(GMA)] was performed, which was used as a matrix for further functionalization. In order to change the hydrophobic character of the [P(EMA)-*co*-(GMA)] to a more hydrophilic one, the oligomeric chain was functionalized with ETA, Xyl or Orn. Synthesis of the [P(EMA)-*co*-(GMA)] and its further modification with moieties containing –OH and –NH_2_ groups allowed us to obtain the products in the form of white or slightly yellow solid powders. The addition of water to the modified [P(EMA)-*co*-(GMA)] enabled the formation of HGs with very good water absorption properties ranging from approx. 120 to 420 wt.% of the oligomer mass. The modified [P(EMA)-*co*-(GMA)] oligomers in the form of HGs were used for the immobilization of MB, which was used as a model compound to study its release. The results show that the release of MB loaded onto the HG is highest and fastest during the first 6 h, and next, that the release is reduced until it reaches the equilibrium with the solution, which was observed during 48 h of the experiment.

Based on the cell culture experiment it may be concluded that only [P(EMA)-*co*-(GMA)] and [P(EMA)-*co*-(GMA)]-Xyl have the potential to be used in biomedical applications as drug carriers. The results indicate that the obtained oligomers can be used in the future to develop materials for the controlled, prolonged release of hydrophilic active substances.

## Figures and Tables

**Table 1:** Average molar mass determinations for the methacrylate oligomer derivatives obtained by size-exclusion chromatography.

	Sample			
Value	[P(EMA)-*co-*(GMA)]	[P(EMA)-*co-*(GMA)]-Xyl	[P(EMA)-*co-*(GMA)]-ETA(2)	[P(EMA)-*co-*(GMA)]-Orn(2)
Average molar mass (Mn)	436.50	615.80	473.20	686.67
Weighted average molar mass (Mw)	478.70	721.78	543.91	783.37
Dispersity index (*Ð*)	1.10	1.17	1.14	1.14

**Table 2:** Water uptake of the modified [P(EMA)-*co*-(GMA)] and their derivatives.

Sample	*m_dried_* (mg)	*m_swollen_* (mg)	*SW*
[P(EMA-*co*-GMA)]-ETA(**1**)	1000	4687	3.70
[P(EMA-*co*-GMA)]-Orn(**1**)	1000	2257	1.25
[P(EMA-*co*-GMA)]-Xyl	1000	3475	2.47
[P(EMA-*co*-GMA)]-ETA(**2**)	1000	5149	4.15
[P(EMA-*co*-GMA)]-Orn(**2**)	1000	4584	3.58

## Data Availability

Not applicable.

## Supplementary Materials

The following are available online, Figure S1: ^13^C NMR spectrum of [P(EMA)-*co*-(GMA)], Figure S2: ^1^H NMR spectrum of [P(EMA)-*co*-(GMA)]-ETA(1), Figure S3: ^1^H NMR spectrum of [P(EMA)-*co*-(GMA)]-ETA(2), Figure S4: ^1^H NMR spectrum of [P(EMA)-*co*-(GMA)]-Orn(2), Figure S5: 1H NMR spectrum of [P(EMA)-*co*-(GMA)]-Xyl, Table S1: Molecular mass distribution for [P(EMA)-*co*-(GMA)], Table S2: Molecular mass distribution for [P(EMA)-*co*-(GMA)]-Xyl, Table S3: Molecular mass distribution for [P(EMA)-*co*-(GMA)]-ETA(2), Table S4: Molecular mass distribution for [P(EMA)-*co*-(GMA)]-Orn(2), Table S5: Determination of epoxy groups for [P(EMA)-*co*-(GMA)] using hydrochloric acid-acetone-standing method.

## Sample Availability

Samples of the compounds and numerical raw data are available from the authors.
