# Supplementary material for: Microwave-Assisted Synthesis of Modified Glycidyl Methacrylate–Ethyl Methacrylate Oligomers, Their Physico-Chemical and Biological Characteristics
Source: Molecules. 2022 Jan 6;27(2):337. doi: 10.3390/molecules27020337 (PMC8779268; doi:10.3390/molecules27020337)
Supplement: Supplementary file 1 [file molecules-27-00337-s001.zip › molecules-1521776-supplementary.pdf]

Supplementary Materials

Microwave-Assisted Synthesis of Modified Glycidyl Methacrylate-Ethyl Methacrylate Oligomers, Their Physico-Chemical and Biological Characteristics

Adam Chyzy [1], *Damian Pawelski* [1], *Vladyslav Vivcharenko* [2], *Agata Przekora* [2], *Michael Bratychak* [3], *Olena Astakhova* [3], *Joanna Breczko* [4], *Pawel Drozdzal* [5] *and Marta E. Plonska-Brzezinska* [1,*]

[1] Department of Organic Chemistry, Faculty of Pharmacy with the Division of Laboratory Medicine, Medical University of Bialystok, Mickiewicza 2A, 15-222 Bialystok, Poland; adam.chyzy@umb.edu.pl (A.Ch.); damian.pawelski@umb.edu.pl (D.P.); marta.plonska-brzezinska@umb.edu.pl (M.E.P.-B.)
[2] Chair and Department of Biochemistry and Biotechnology, Medical University of Lublin, Chodzki 1, 20-093 Lublin, Poland; vlad.vivcharenko@gmail.com (V.V.); agata.przekora@umlub.pl (A.P.)
[3] Lviv Polytechnic National University, 12, St. Bandera str., 79013 Lviv, Ukraine; mbratychak@gmail.com (M.B.); olena.brat@gmail.com (O.A.)
[4] Faculty of Chemistry, University of Bialystok, Ciolkowskiego 1k, 15-245 Bialystok, Poland; j.luszczyn@uwb.edu.pl (J.B.)
[5] Department of Structural Biology of Prokaryotic Organisms, Institute of Bioorganic Chemistry, Polish Academy of Sciences, Z. Noskowskiego St. 12/14, 61-704 Poznan, Poland; pdrozdzal@ibch.poznan.pl (P.D.)

* Correspondence: marta.plonska-brzezinska@umb.edu.pl (M.E.P.-B.); Tel.: +4885-748-5687

Figure S1. ^{13}C NMR spectrum of [P(EMA)-*co*-(GMA)]. ^{13}C NMR (176 MHz, DMSO-d_6), δ [ppm] : 176.9 and 175.8 (>C=O), 79.2 and 65.7 (>C-OR), 60.5, 53.5, 48.6, 48.5, 18.4, 16.5, 13.5.

Figure S2. ^{1}H NMR spectrum of [P(EMA)-*co*-(GMA)]-ETA(**1**). ^{1}H NMR (400 MHz, DMSO-d_6), δ [ppm] : 0.76 and 0.92 (br, backbone CH_3), 1.17 (br, ethyl ester CH_3), 1.77-1.89 (m, backbone CH_2), 2.59 (br, -CH_2-NH-CH_2-), 3.34-3.37 (m, O-CH_2-CH<), 3.76 (br, CH_2-OH), 3.96 (br, CH_2 in Et).

Figure S3. ^{1}H NMR spectrum of [P(EMA)-*co*-(GMA)]-ETA(**2**). ^{1}H NMR (400 MHz, DMSO-d_6), δ [ppm] : 0.76 and 0.92 (br, backbone CH_3), 1.12-1.17 (m, ethyl ester CH_3), 1.77-1.89 (m, backbone CH_2), 2.59 (br, -CH_2-NH-CH_2-), 3.34-3.37 (m, O-CH_2-CH<), 3.75 (br, CH_2-OH), 3.97 (br, CH_2 in Et).

Figure S4. ^{1}H NMR spectrum of [P(EMA)-*co*-(GMA)]-Orn(**2**).

Figure S5. ^{1}H NMR spectrum of [P(EMA)-*co*-(GMA)]-Xyl.

Table S1. Molecular mass distribution for [P(EMA)-*co*-(GMA)].

Table S2. Molecular mass distribution for [P(EMA)-*co*-(GMA)]-Xyl.

Table S3. Molecular mass distribution for [P(EMA)-*co*-(GMA)]-ETA(**2**).

Table S4. Molecular mass distribution for [P(EMA)-*co*-(GMA)]-Orn(**2**).

Table S5. Determination of epoxy groups for [P(EMA)-*co*-(GMA)].

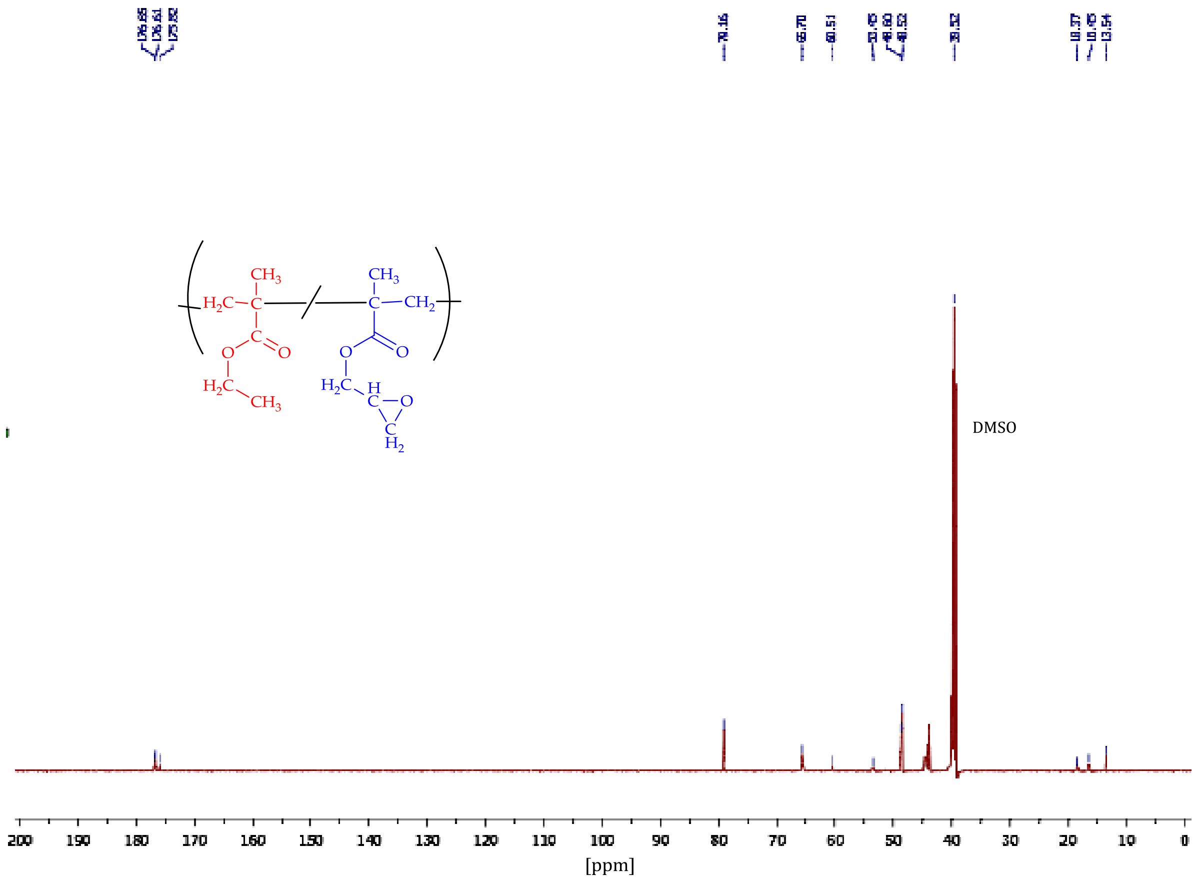

Figure S1. ^{13}C NMR spectrum of [P(EMA)-*co*-(GMA)]. ^{13}C NMR (176 MHz, DMSO-d_6), δ (ppm): 176.9 and 175.8 (>C=O), 79.2 and 65.7 (>C-OR), 60.5, 53.5, 48.6, 48.5, 18.4, 16.5, 13.5.

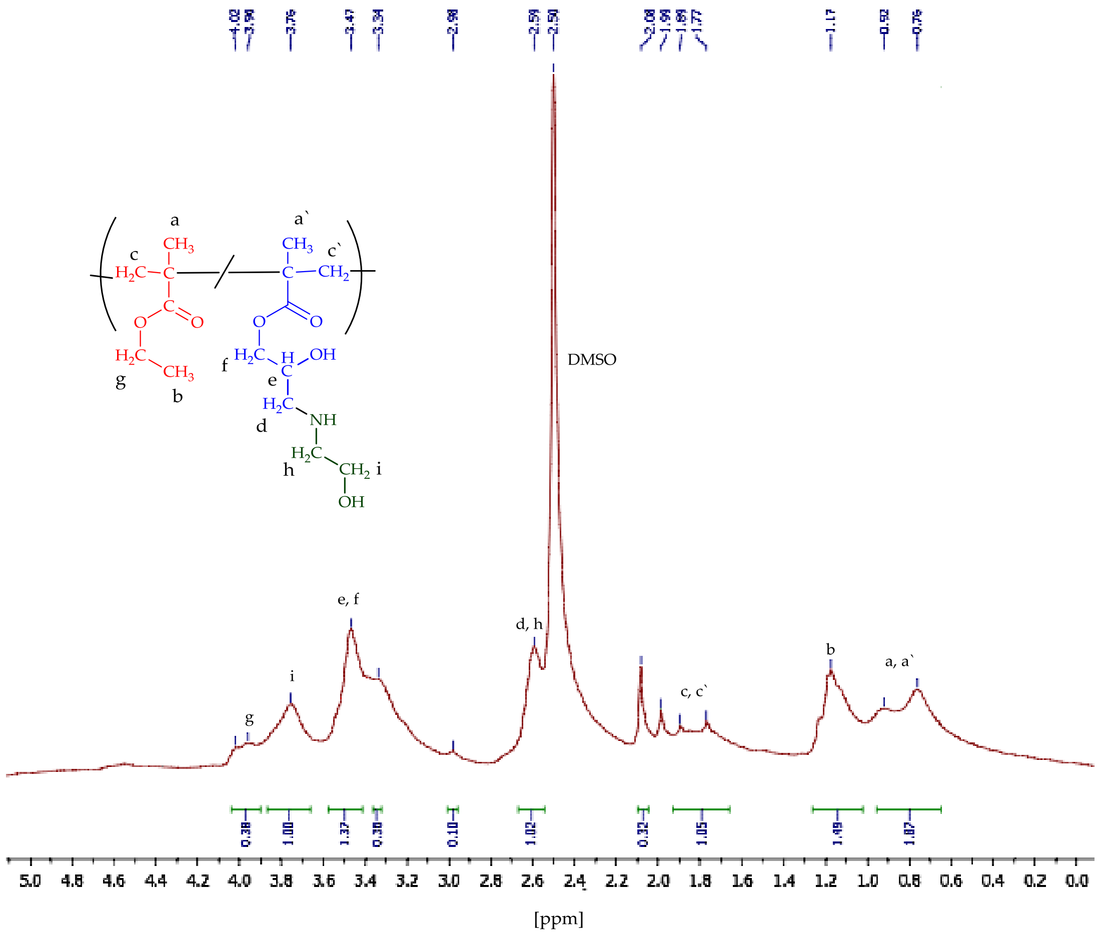

Figure S2. ^1H NMR spectrum of [P(EMA)-*co*-(GMA)]-ETA(**1**). ^1H NMR (400 MHz, DMSO-d_6), δ (ppm): 0.76 and 0.92 (br, backbone CH_3), 1.17 (br, ethyl ester CH_3), 1.77-1.89 (m, backbone CH_2), 2.59 (br, -CH_2-NH-CH_2-), 3.34-3.37 (m, O-CH_2-CH<), 3.76 (br, CH_2-OH), 3.96 (br, CH_2 in Et).

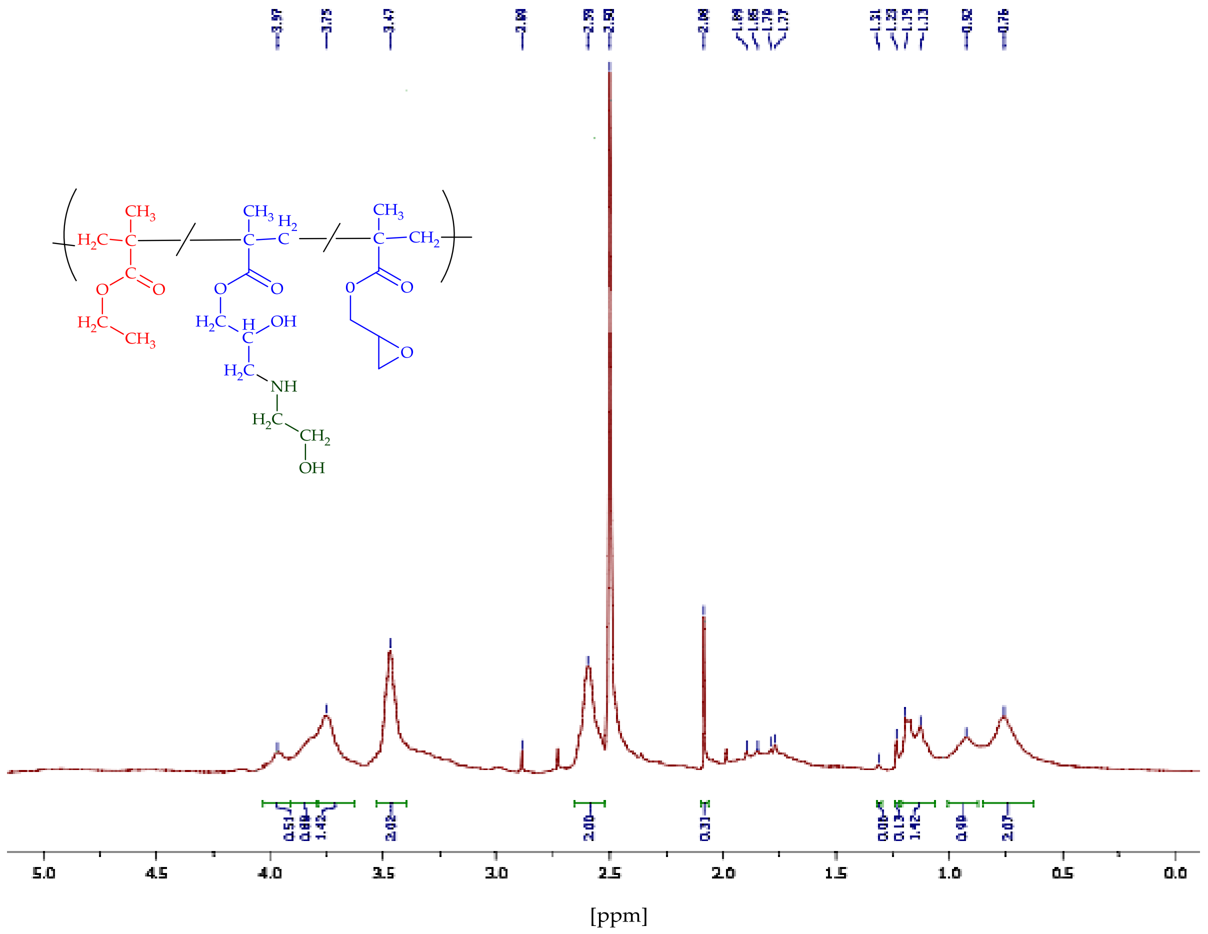

Figure S3. ¹H NMR spectrum of [P(EMA)-*co*-(GMA)]-ETA(**2**). ¹H NMR (400 MHz, DMSO-d_6), δ (ppm): 0.76 and 0.92 (br, backbone CH_3), 1.12-1.17 (m, ethyl ester CH_3), 1.77-1.89 (m, backbone CH_2), 2.59 (br, -CH_2-NH-CH_2-), 3.34-3.37 (m, O-CH_2-CH<), 3.75 (br, CH_2-OH), 3.97 (br, CH_2 in Et).

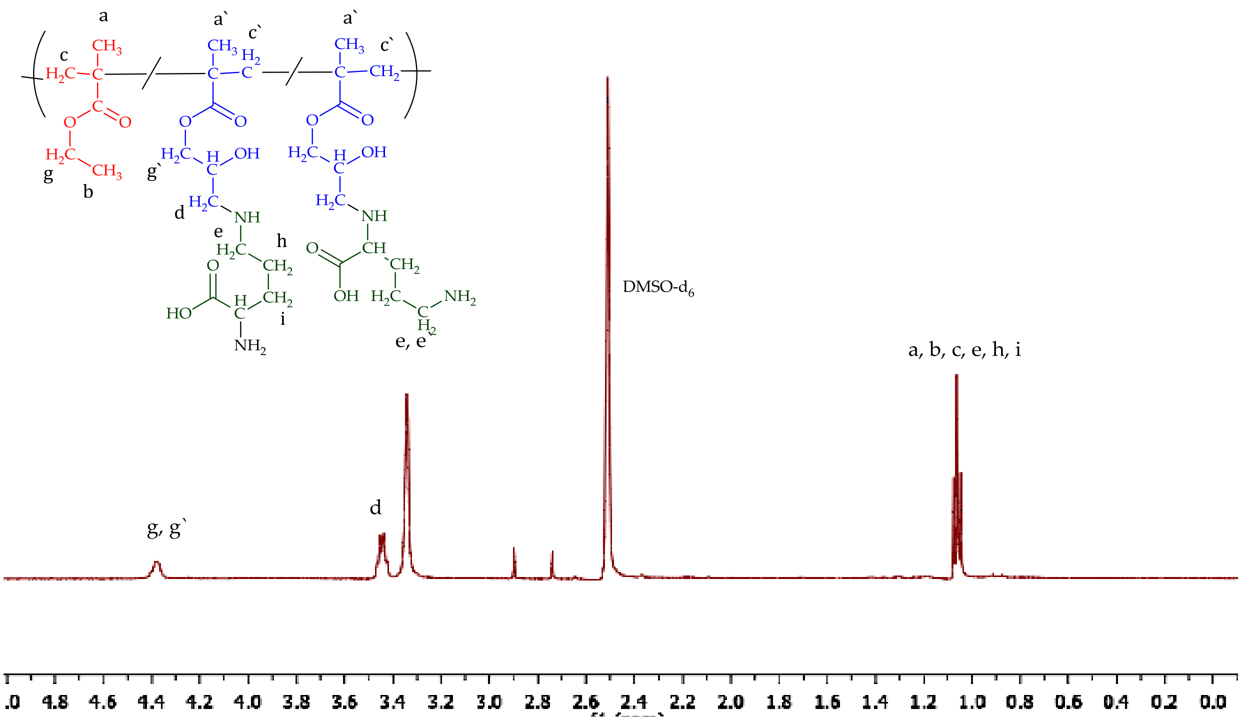

Figure S4. ^{1}H NMR spectrum of [P(EMA)-*co*-(GMA)]-Orn(**2**).

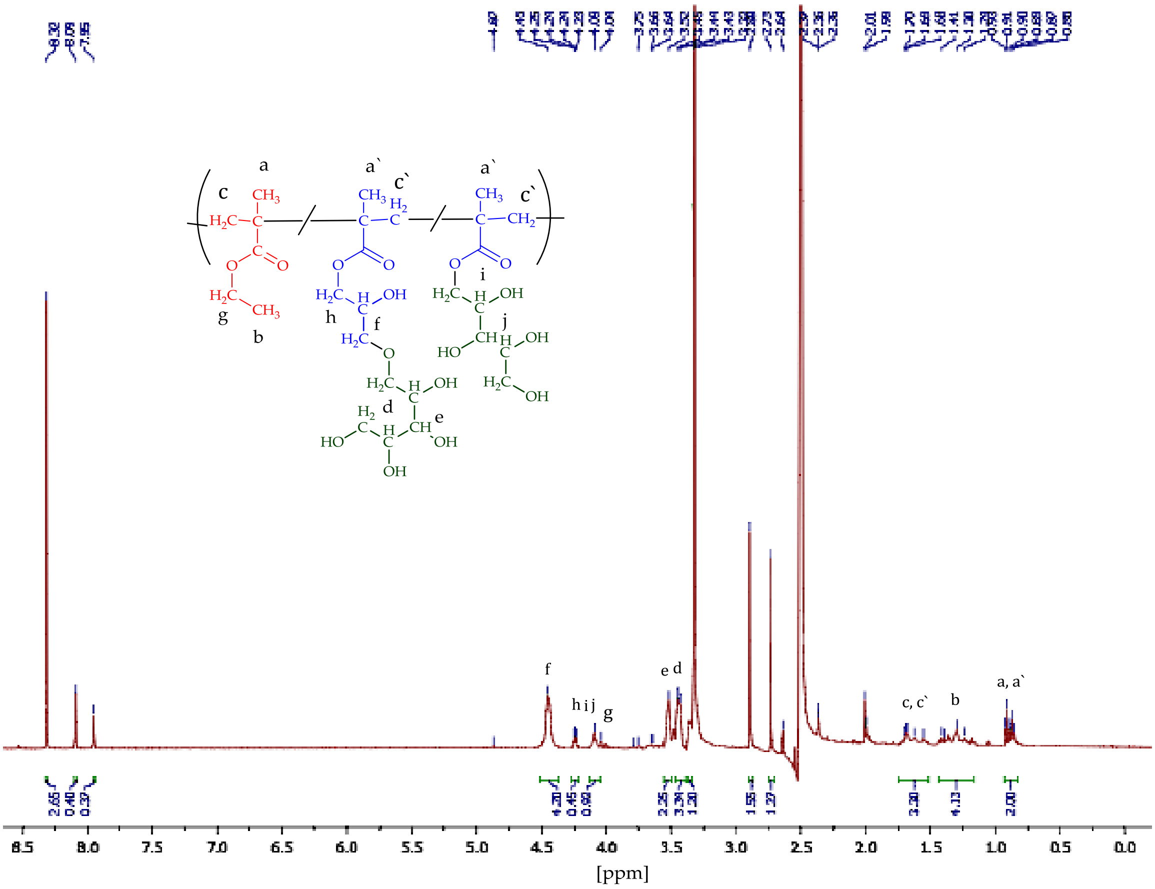

Figure S5. ^{1}H NMR spectrum of [P(EMA)-*co*-(GMA)]-Xyl.

Table S1. Molecular mass distribution for [P(EMA)-*co*-(GMA)].

**Mass range (Da)**	**[P(EMA)-*co*-(GMA)]**
	Percentage (%)
1482.8-1202.2	0.50
1202.2-1004.3	0.77
1004.3-601.2	4.18
601.2-300.8	86.84
300.8-234.4	7.71

Table S2. Molecular mass distribution for [P(EMA)-*co*-(GMA)]-Xyl.

**Mass range (Da)**	**[P(EMA)-*co*-(GMA)]-Xyl**
	Percentage (%)
2754.7-2007.2	0.38
2007.2-1004.1	5.61
1004.1-601.3	28.34
601.3-270.5	65.67

Table S3. Molecular mass distribution for [P(EMA)-*co*-(GMA)]-ETA(**2**).

**Mass range (Da)**	**[P(EMA)-*co*-(GMA)]-ETA**
	Percentage (%)
2664.4-2001.2	0.01
2001.2-1004.2	2.45
1004.2-601.3	9.91
601.3-217.1	87.64

Table S4. Molecular mass distribution for [P(EMA)-*co*-(GMA)]-Orn(**2**).

**Mass range (Da)**	**[P(EMA)-*co*-(GMA)]-Orn**
	Percentage (%)
2560.1-2007.4	0.50
2007.4-1004.2	5.72
1004.2-601.3	57.75
601.3-217.1	36.03

Table S5. Determination of epoxy groups for [P(EMA)-*co*-(GMA)] using hydrochloric acid-acetone-standing method.

Sample	Mass of oligomer (g)	Volume of titrant (mL)	L_{ep} (%)
blank	0	30.55	-
1	0.1999	18.40	26.25
2	0.2003	18.70	25.44
3	0.2003	18.30	26.30
